# Use of antipsychotics with tamoxifen

**DOI:** 10.1192/j.eurpsy.2021.1154

**Published:** 2021-08-13

**Authors:** H. Ghabi, M. Karoui, F. Ellouz

**Affiliations:** 1 Department Of Psychiatry G, Razi hospital, Manouba, Tunisia; 2 Department Of Psychiatry G, razi hospital, manouba, Tunisia; 3 Department Of Psychiatry G, Razi hospital, manouba, Tunisia

**Keywords:** tamoxifen, Antipsychotics, interaction

## Abstract

**Introduction:**

Bipolar disorder is a frequent and serious psychiatric disease. Antipsychotics are habitually required for its management especially during an acute manic episode. The association of cancer with bipolar disorder may impact psychiatric management. The choice of the adequate antipsychotic drug remains a challenge in this case. The clinical benefit of tamoxifen is obtained after the hepatic metabolism with cytochrome P450 2D6 which generates endoxifen, the potent metabolite of tamoxifen. Evidence has emerged that antipsychotics may potentially inhibit the CYP2D6. Study data supporting this interaction are rare.

**Objectives:**

In this work, we aimed to illustrate the modalities of care of bipolar disorder in a patient receiving tamoxifen.

**Methods:**

Presentation of a clinical case of a patient treated by Tamoxifen for her breast cancer and who was admitted in our department for acute mania with psychotics features, followed by a literature review.

**Results:**

A 53-year-old woman with past history of breast cancer diagnosed in 2018, treated with lumpectomy and radiation, followed by tamoxifen. She has been admitted in 2019 in our department for an acute mania with psychotics features. Olanzapine was prescribed with good clinical evolution. The psychiatric and oncologic status of the patient was stable after one year under tamoxifen and olanzapine.

**Conclusions:**

Psychiatrists must be aware that some of the prescribed medications co-administered with tamoxifen interfere with the CYP2D6 function, which may potentially increase the risk of breast cancer recurrence. A close collaboration between psychiatrists and oncologists is required to adapt therapeutic protocols.

